# Is injectable platelet rich fibrin beneficial in managing intracapsular temporomandibular disorders? A systematic review and meta-analysis

**DOI:** 10.1186/s12903-025-07331-1

**Published:** 2025-12-09

**Authors:** Mariam M. Bahgat, Nourhan M. Aly

**Affiliations:** 1 Department of Prosthodontics, Faculty of Dentistry, Alexandria University, Azarita, Alexandria, 21526 Egypt; 2 Department of Pediatric Dentistry and Dental Public Health, Faculty of Dentistry, Alexandria University, 21526 Alexandria , Egypt

**Keywords:** Injectable Platelet Rich Fibrin, Temporomandibular disorders, Maximum mouth opening, Visual analogue scale, Meta-analysis

## Abstract

**Objective:**

To highlight the current knowledge of the effectiveness of injectable platelet rich fibrin (i-PRF) in the management of temporomandibular joint internal derangement (TMJ-ID) and temporomandibular joint osteoarthritis (TMJ-OA).

**Methodology:**

A “Population, Intervention, Comparison, Outcome” (PICO) strategy was implemented by performing a systematic search through Cochrane databases, PubMed/MEDLINE, and Google Scholar from their commencement to November 2024. Randomized clinical trials (RCTs) addressing the management of TMJ-ID and TMJ-OA with i-PRF were included. Two reviewers independently evaluated the suitability of the RCTs, followed by data extraction.

**Results:**

The electronic search revealed 274 articles, but only 6 studies were deemed suitable for selection, with a total of 270 subjects; 75% were females, and 25% were males. Meta-analysis revealed a significant reduction in pain levels with i-PRF compared to controls (pooled SMD = − 2.16; 95% CI = − 3.43 to − 0.89; *P* < 0.001). Significant improvement was also observed in maximum mouth opening (SMD = 2.46; 95% CI = 0.56 to 4.36; *P* = 0.02). i-PRF significantly enhanced lateral mandibular movements compared with controls (SMD = 1.35; 95% CI = 0.03 to 2.67; *P* = 0.048), whereas no significant difference was found for protrusive movements (SMD = 1.76; 95% CI = − 0.97 to 4.48; *P* = 0.11). Improvements in joint sounds and disc position were qualitatively reported in smaller subsets of studies. Substantial heterogeneity (I² = 83–93%) was observed across outcomes, reflecting variability in study design and treatment protocols.

**Conclusion:**

Current evidence suggests that i-PRF provides improvements in TMJ pain, joint sounds, and mandibular movements for up to 12 months. However, more RCTs are needed.

## Introduction

Temporomandibular disorders (TMDs) are the most common orofacial pain conditions of non-dental origin that affect the muscles and the temporomandibular joint (TMJ) [1]. TMDs can be categorized as extracapsular or intracapsular disorder. In the extracapsular disorders, it comprises masticatory muscle disorders or myogenous disorders involving myofascial pain. While in intracapsular one, it covers arthrogenous disorders involving TMJ internal derangement (TMJ-ID) where an abnormal disc-condyle relationship exists, and osteoarthritis (TMJ-OA) affecting all the TMJ structures including the articular disc, subchondral bone, ligaments, capsule, synovium, and retrodiscal tissues [2–5]. 

The salient signs and symptoms of intracapsular TMDs are joint clicking, pain during mandibular movements and limitation of range of motion [3–5]. Pain in intracapsular TMDs is localized in the periauricular area due to mechanical abuse of the healthy joint structures and impingement of tissues leading to inflammation of the retrodiscal tissues. Understanding the symptoms related to TMDs and their negative impact on the patient’s welfare makes the treatment of this disorder essential.

Management of patients suffering from TMDs should aim to relieve joint pain and improve dysfunction with the prevention of the disease progression. Throughout the years, treatment modalities of TMDs ranged from non-surgical approaches to surgical ones [6–12]. However, conservative as well as minimally invasive techniques, including physiotherapy, drug therapy, oral appliances, intra-articular injections, or a combination of these modalities [7, 9], should be considered as a first-line approach prior to any invasive interventions. Nevertheless, recent research supports the positive effect of certain therapies such as the injection of autologous blood or platelet concentrates (PC) in cases of TMJ-ID and TMJ-OA [13–31]. 

PC derived through the centrifugation of a blood sample, contains various growth factors with anti-inflammatory effects and healing enhancing properties; hence, stimulate cell proliferation and tissue repair [32–35]. The theory about PC effect is the in-situ release of autogenous growth factors which promote a revolution in regenerative medicine.

Marx et al. [34] introduced the platelet-rich plasma (PRP) as the first generation of PC. The main advantage of PRP is its fluid nature, allowing it to be used as an injectable regenerative agent for osteoarthritic joints. Several studies have documented its beneficial effect in the treatment of intracapsular TMDs. However, PRP has two main disadvantages where it requires the addition of an anticoagulant and the complex preparation procedure.

In 2000, a second generation of PC, the platelet rich fibrin (PRF), was developed by Choukroun et al. [35]. PRF is a fibrin-rich gel derived from a venous blood sample that is collected in plain tubes with no anticoagulant, then immediately centrifuged at a fixed speed for 10 min in a single step. As a result of this process, a fibrin-rich gel with aggregated platelets is formed between the red blood cells and platelet poor plasma. Nearly two decades after preparing PRF, Choukroun et al. [36] introduced a new low-speed centrifugation protocol where thrombin and liquid fibrinogen are not yet turned into fibrin resulting in a liquid PRF. This injectable PRF (i-PRF) is rich in platelets, white blood cells, and growth factors which accelerate new bone formation, cartilage repair and tissue healing. Since then, the i-PRF has been injected into joint spaces like the PRP, but with a quicker preparation procedure and longer growth factors in situ release time [36–38]. 

Therefore, this systematic review was conducted to investigate the RCTs available in the literature addressing the treatment outcomes of i-PRF on patients with TMJ-ID and TMJ-OA. Our focused question was “Is i-PRF effective in the management of intracapsular TMDs regarding pain relief and increased mouth opening?” In formulating our review question, we put into consideration identifying the problem “intracapsular TMDs”, intervention “i-PRF”, comparison “any other intervention,” and outcome “treatment”.

Thus, the purpose of this systematic review is to assess the efficacy of i-PRF in the management of TMJ-ID and TMD-OA. Furthermore, it highlights the preparation and injection protocols of i-PRF in the TMJ.

## Materials and methods

A PICO strategy was formulated using an electronic search through Cochrane and PubMed/MEDLINE databases in addition to Google Scholar, focusing on randomized clinical trials (RCTs) investigating the management of TMDs with i-PRF. These databases were searched from their inception to November 2024.

The keywords used while searching for relevant studies included specific Medical Subject Headings (MeSH) terms including “platelet rich fibrin” AND “temporomandibular disorders” AND “pain” AND “mouth opening”. Published RCTs that answer the main study question assessing the use of i-PRF in patients suffering from TMJ-ID and TMJ-OA as defined by the DC/TMDs, and RCTs assessing the treatment outcomes of i-PRF in patients with limited MMO were included. Studies without control or comparative arm, retrospective studies, narrative reviews, and animal studies were excluded.

Two reviewers (M.B.M and N.M.H) independently assessed the eligibility of the retrieved electronic titles and abstracts, then evaluated the potentially relevant full-text articles. Subsequently, data was extracted from the selected eligible articles. The agreement between both reviewers was appraised by the Cohen’s Kappa coefficient. Disagreements in the assessments in study selection and data extraction were settled by direct discussion among reviewers. Qualitative data analysis was conducted by applying descriptive methods.

### Meta-analysis

Meta-analysis was done using Meta-Analysis Online (https://metaanalysisonline.com/). The heterogeneity of the included studies was judged using I^2^ values and Q-test. Forest plots with random-effects models were used in the current study due to the high degree of heterogeneity encountered across all outcomes. The estimates were pooled using inverse variance random-effects model and demonstrated as standardized mean difference (SMD) and 95% confidence intervals (CIs). A *p*-value of less than 0.05 was deemed statistically significant.

### Risk of bias

Appraisal of the RCTs was done with the Cochrane Collaboration’s tool for assessing risk of bias [39].

## Results

**Fig. 1:**
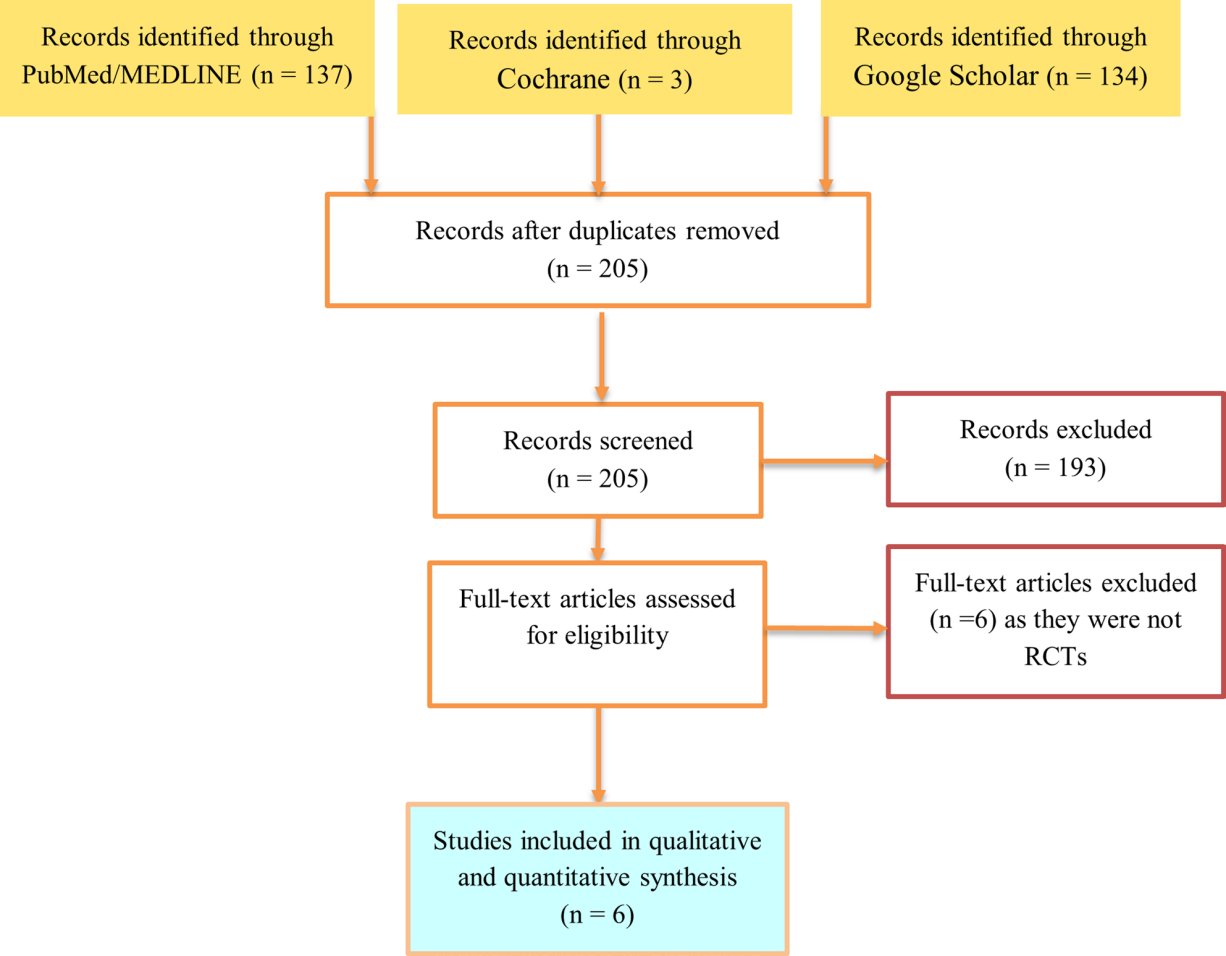
PRISMA Flow Chart for identification and selection of eligible studies

The systematic search identified 137 studies from PubMed, 134 articles from Google Scholar, and 3 studies from the Cochrane library. Of the 274 screened articles and after duplicate removal, the full texts of 12 articles were assessed, and only 6 articles [25–30] were considered eligible as they fulfilled the inclusion criteria. Thus, they were included for data extraction (Fig. 1). Excluded articles with reasons are listed in Table 1.

**Table 1:** Excluded studies

Exclusion criteria	Articles
Not RCT	Torul et al. 2019 [22], Yuce et al. 2020 [23], Albilia et al. 2020 [15], González et al. 2021 [16], Manafikhi et al. 2022 [24], Vingender et al. 2023 [31]

A total of 270 subjects participated in the studies reviewed. In five studies [25–27, 29, 30], the gender of patients was identified (201 females, 55 males). In all the included studies [25–30], sample selection was not gender-based, with an interesting finding in Işik et al. study [27] where only 3 participants were males. Four studies [25–27, 29, 30] identified the age range of the participants. However, one study [28] did not identify the age range but mentioned that the average age in each study group. Demographic data are presented in Table 2.

**Table 2:** Demographic data of the included studies

Author/Year	Sample size	Female No. (%)	Male No. (%)	Age Range (years)
Karadayi et al. 2021 [25]	36	19 (53%) (i-PRF group:10; Control group:9)	17 (47%) (i-PRF group: 8; Control group:9)	20–64 (mean age = 39.82)
Ghoneim et al. 2022 [26]	40	29(72.5%) (i-PRF group:16; Control group:13)	11(27.5%) (i-PRF group:4; Control group:7)	18–55 (i-PRF group: 44.67 ± 12.13; control group: 45.72 ± 13.12)
Işik et al. 2022 [27]	*36*	*33(92%)* *(i-PRF group:16;* *Control group:17)*	*3(8%)* *(i-PRF group:2;* *Control group:1)*	*≥* *18* *(i-PRF group: 44.67 ± 12.13; control group: 45.72 ± 13.12)*
Sharma et al. 2023 [28]	*14*	Not identified	Not identified	*20–50*
Işik et al. 2023 [29]	*76*	*69(90.8%)* *(i-PRF group:34;* *Control group:35)*	*7(9.2%)* *(i-PRF group:4;* *Control group:3)*	*≥* *18* *(i-PRF group: 47.2 ± 9.1; control group: 46.8 ± 10.2)*
Kumar et al. 2024 [30]	*68*	*51(75%)* *(i-PRF group:25;* *Control group:26)*	*17(25%)* *(i-PRF group:9;* *Control group:8)*	*18–60* *(i-PRF group: 42.5 ± 8.2; control group: 41.8 ± 7.5)*
*Total*	***270*** ***256*** *(with gender identified)*	***201 (78.5*** **%)**	***55 (21.5%)***	

All the included studies [25–30] investigated the effectiveness of i-PRF on joint pain and MMO. All studies [25–30] used Visual Analogue Scale (VAS) to assess pain. One study [25] applied Helkimo clinical dysfunction scoring and TMJ clicking was assessed in two studies [25, 27]. Four studies [25–29] noted the effect of i-PRF on the lateral excursions while three studies [27–29] evaluated its effect on the protrusive movements as well. Only one study [28] evaluated the disc position and joint effusion on MRI after the treatment. A synopsis of the characteristics of the studies reviewed is provided in Table 3, and findings of the studies on TMD pain, MMO, and reported side effects are grouped in Table 4. Furthermore, this meta-analysis assessed the effects of i-PRF on various TMJ outcomes involving pain, MMO, protrusive, and lateral movements.

**Table 3:** Summary of the characteristics of the included studies

Author/Year	Sample size	TMDs	i-PRF group	control/comparative group(s)	Injection Site(s)	Use of US	Injection Vol./Freq.	Follow-up periods	Outcomes
Karadayi et al. 2021 [25]	36	TMJ-ID (Wilkes stage > 3)	Arthrocentesis + i-PRF (*n* = 18)	Arthrocentesis (Lactated Ringer solution) (*n* = 18)	Superior joint space	No	2 ml/one time	Day 0,10,30,90	VAS (0–10) Helkimo Clinical Dysfunction Score MMO
Ghoneim et al. 2022 [26]	40	TMJ-ID (DDr)	Arthrocentesis + i-PRF (*n* = 20)	Arthrocentesis (Lactated Ringer solution) (*n* = 20)	Superior joint space	No	1.5 ml/one time	Week 0,7,21,24	VAS (0–10) Clicking MMO, lateral excursions (mm)
Işik et al. 2022 [27]	*36*	*TMJ-OA*	*Arthrocentesis* *+ i-PRF* (*n* = 18)	Arthrocentesis (saline solution) (*n* = 18)	*Superior joint space*	*No*	*1 ml/4 times (0*,*1*,*2*,*3w)*	*Month* *0*,*1*,*2*,*3*,*6*,*12*	*VAS (0–10)* *MMO*,* lateral & protrusive movements (mm)*
Sharma et al. 2023 [28]	*14*	*TMJ-ID* *(Wilkes stage 1–4)*	*Arthrocentesis (Lactated Ringer solution) + i-PRF* (*n* = 7)	*Arthrocentesis (Lactated Ringer solution) + PRP* (*n* = 7)	*Superior joint space*	*No*	*2 ml/6 times* *(0*,*1*,*2*,*3*,*4*,*5 m)*	*Month* *0*,*1*,*2*,*3*,*4*,*5*,*6*,*9*	*VAS (0–10)* *MMO*,* lateral & protrusive movements (mm)* *Joint sounds* *MRI (Disc position*,* JE)*
Işik et al. 2023 [29]	*76*	*TMJ-ID* *(DDWor)*	*Arthrocentesis* *+ i-PRF* (*n* = 38)	Arthrocentesis (saline solution) (*n* = 38)	*Superior joint space*	*No*	*1 ml/4 times (0*,*1*,*2*,*3w)*	*Month* *0*,*1*,*2*,*3*,*6*,*12*	*VAS (0–10)* *MMO*,* lateral & protrusive movements (mm)*
Kumar et al. 2024 [30]	*68*	*Unspecified* *(TMJ pain)*	*i-PRF* (*n* = 38)	*No active treatment* (*n* = 38)	*Superior joint space*	*No*	*1 ml/twice* *(0*,*1 w)*	*Week* *0*,*6*,*12*,*24*	*VAS (0–10)* *MMO (mm)* PROs QoL (*Questionnaires)*

*TMDs* Temporomandibular Disorders, *TMJ-ID *Temporomandibular Joint Internal Derangement, *TMJ-OA *Temporomandibular Joint Osteoarthritis, *DDr* Reducible Disc Displacement, *i-PRF *Injectable Platelet Rich Fibrin, *PRP *Platelet Rich Plasma, *DDWor *Disc Displacement without reduction, *US *Ultrasound, *w *weeks, *VAS* Visual Analog Scale, *MMO* Maximum Mouth Opening, *PROs QoL* Patient-Reported Outcomes on Quality of Life and Satisfaction

**Table 4:** Findings of the included studies on TMD pain, MMO, and reported side effects

Author/Year	Effect on TMD Pain	Effect on MMO	Other Findings	Side Effects	Conclusions
Karadayi et al. 2021 [25]	٠ The mean VAS scores after 30 days showed a statistically significantly lower mean score compared to baseline (p < 0.001). ٠ Difference between groups was statistically significant at the follow-ups.	٠ The mean MMO after 1 m & 3 m showed statistically significantly higher mean value than baseline in both groups. ٠ After 30 days, there was a statistically significant difference between both groups, where i-PRF group showed higher MMO than the control group. ٠ At 3 m, no statistically significant difference was noted between both groups.	٠ After 10 days, an insignificant decrease in HCDS was noted in each group. However, the difference between both groups was statistically significant. ٠ There was a statistically significant decrease in HCDS after 30 days with statistically significant difference between the 2 groups.	None	٠ The use of i-PRF with arthrocentesis showed better results than arthrocentesis alone. ٠ Multiple PRF injections might be applied for severe TMDs.
Ghoneim et al. 2022 [26]	٠ Within each group, a significant relief of pain was noted at the follow-ups. ٠ There was a statistically significant difference (p < 0.001) between the two groups along the evaluation periods.	٠ After 1 w, the MMO was significantly increased in both groups (p = 0.032). However, the i-PRF group showed higher values than the control group. ٠ Arthrocentesis + i-PRF outperformed arthrocentesis alone with a statistically significant difference (p < 0.05).	٠ TMJ clicking was assessed showing a statistically significant difference (p < 0.05) between both groups at the follow-up periods. ٠ Statistically significant differences between clicking scores within each group was noted. ٠ In the i-PRF group, TMJ clicking disappeared in 75% of the patients after 7 days post injection (p < 0.001). ٠ The measurements of lateral movements showed a statistically significant increase within each group over time. The difference between both groups was statistically significant.	In the i-PRF group, 2 patients showed transient inability to occlude in maximum intercuspation in the 1st 48 h post- injection.	٠ Additional i-PRF injections after arthrocentesis are of more effectiveness than arthrocentesis alone in the management of TMJ-ID. ٠ The intracapsular injection of i-PRF is a low-risk method with promising outcomes regarding relief of pain, TMJ clicking & improving range of mandibular movements.
Işik et al. 2022 [27]	٠ A decrease in pain levels was noted in both groups, with a statistically significant difference. ٠ A significant increase in pain levels was observed only in the control group from the 6th to 12th m (p < 0.001). Difference between groups was statistically significant at follow-up periods.	٠ In the i-PRF group, the MMO increased postoperatively, and this improvement was maintained up to the 12th m. ٠ Difference between groups was statistically significant during follow-up periods, where the i-PRF group showed higher values than the control group. ٠ A significant decrease in MMO was noted in the control group from the 6th to 12th m postoperatively (p < 0.001).	٠ In the i-PRF group, there was an increase in the range of lateral and protrusive movements up to 12 m with a statistically significant differences at the 1 st and 2nd m postoperatively (p < 0.001), and no significant differences from the 2nd to the 12th m. ٠ As for functional jaw movements, the difference between both groups was statistically significant. ٠ The range of excursive movements in the i-PRF group were significantly higher than that in the control group during follow-up periods. ٠ The range of lateral and protrusive movements decreased significantly in the control group from the 6th to 12th m postoperatively (p < 0.001)	None	Intracapsular injection of i-PRF after arthrocentesis should be preferred as it reduces pain levels & improves jaw movements up to 12 m.
Sharma et al. 2023 [28]	٠ The mean of VAS score showed a statistically significant difference between both groups at 1 week after the 1st injection and 1 week after the 2nd injection. While at 1 week after the 3rd, 4th, 5th, and 6th injections and at the 9th month follow–up, no statistical difference (p = 0.450, 0.805, 0.338, 0.509, and 0.509, respectively) was found. ٠ In the i-PRF group, no statistically significant reduction was noted for 1st and 2nd follow up followed by a significant reduction till 9 months (p < 0.001).	٠ The intragroup comparison of MMO from baseline to various follow up revealed that in PRP group and i-PRF group II statistically significant change was found in the 1st month (p = 0.015 and p = 0.008 respectively). ٠ A statistically significant difference was observed between the groups 3rd injection onward (p < 0.001).	٠ Both groups demonstrated statistically significant results where i-PRF group showed better results as compared to PRP group regarding the improvement of lateral and protrusive movement.٠ A significant disappearance in TMJ clicking was noted in patients 2 weeks posttreatment and was sustained through the follow-ups. However, no statistically significant difference was noted in the presence of joint sound between the groups. ٠ Disc position, on MRI, had improved toward normal with significant changes in both groups at the 9 month follow up; however, the i-PRF group showed better results. ٠ At the 9 month follow up, the joint effusion was significantly less in both groups	None	i PRF has showed better results regarding the efficacy over PRP across all assessed parameters.
Işik et al. 2023 [29]	٠ Pain on palpation, chewing, and jaw movements decreased significantly on the 1st m postoperatively in both groups. ٠ A significant increase in pain levels was observed only in the control group from the 6th to 12th m (p < 0.001). ٠ Difference between groups was statistically significant at follow-up periods(p < 0.001).	٠ A statistically significant increase in the degree of MMO was noted at the 1st, 2nd, 3rd, and 6th m postoperatively, when compared to preoperative measurements for each group. ٠ The increase in MMO was preserved up to the 12th m in the i-PRF group while a significant decrease in MMO was noted in the control group from the 6th to 12th m postoperatively (p < 0.001). ٠ Difference between groups was statistically significant, where the i-PRF group showed higher values than the control group.	٠ In the i-PRF group, there was an increase in the measurements of contralateral, ipsilateral, and protrusive movements up to 12m with a statistically significant difference at the 1st and 2nd m postoperatively (p < 0.001), and no significant differences from the 2nd to the 12th m. ٠ The measurements of contralateral, ipsilateral, and protrusive movements in the i-PRF group were significantly higher than that in the control group during follow-up periods. ٠ Significant decreases in measurements of jaw movements were noted in the control group from the 6th to 12th m postoperatively (p < 0.001).	None	i-PRF used with arthrocentesis is an effective adjunctive management for DDWor as it relieves clinical symptoms.
Kumar et al. 2024 [30]	٠ Through the follow-ups, the i-PRF group showed a noteworthy reduction in TMJ pain intensity compared to the control group that was statistically significant. (p < 0.001).	٠ At all the follow-ups, a substantial improvement in MMO within the i-PRF group was noted compared to controls (p<0.001).	٠ A notable increase in PROs QoL in the i-PRF group was observed compared to controls (p<0.001) in which the patients receiving i-PRF reported improvements in their overall well-being and treatment satisfaction.	None	i-PRF showed significant efficacy in relieving TMJ pain, improving MMO, and enhancing the patients’ quality of life.

VAS Visual analogue Scale, MMO Maximum Mouth Opening, m months, i-PRF Injectable Platelet Rich Fibrin, HCDS Helkimo clinical dysfunction score, m months, h hours, TMJ Temporomandibular Joint, TMJ-ID Temporomandibular Joint Internal Derangement

### i-PRF Preparation protocol

In all studies [25–30], Choukroun et al. [36] protocol was utilized to prepare i-PRF where un-coated tubes without additives were used for blood collection and then centrifuged at a low speed (700 rpm) for three minutes.

### i-PRF injection protocol and site

In all the included studies [25–30], injections were done under local anesthesia. However, Karadayi et al. [25] and Sharma et al. [28] performed auriculotemporal nerve block while in Ghoneim et al. [26] study, local anesthesia was infiltrated into the joint. In both studies by Işık et al. [27, 29], the exact technique for administration of LA was not mentioned.

Regarding the injection of i-PRF, ultrasound-guided injection was not applied in all the included studies [25–30]. However, skin anatomic landmarks were used to identify the site of injection; the superior joint compartment, where i-PRF was injected 1 cm in front of the tragus and 2 mm beneath the canthus-tragus line.

In 5 studies [25–29], the injection of i-PRF was accompanied by arthrocentesis; however, in one study [30] arthrocentesis was not performed. 

### i-PRF injection volume and frequency

Different volumes of i-PRF were used in the included studies where it ranged from 1 ml to 2 ml. In Karadayi et al. [25] study and Sharma et al. [28] study, 2 ml were injected while Işik et al. [26, 29] and Kumar et al. [30] utilized 1 ml per injection. Ghoneim et al. [26] administrated 1.5 ml of i-PRF. Single i-PRF injection was performed in 2 studies [25, 26], i-PRF was injected twice in one study [30], while 4 injections were applied in 2 studies [27, 29], and 6 injections were administered in one study [28].

### i-PRF effect on TMJ pain

**Fig. 2:**
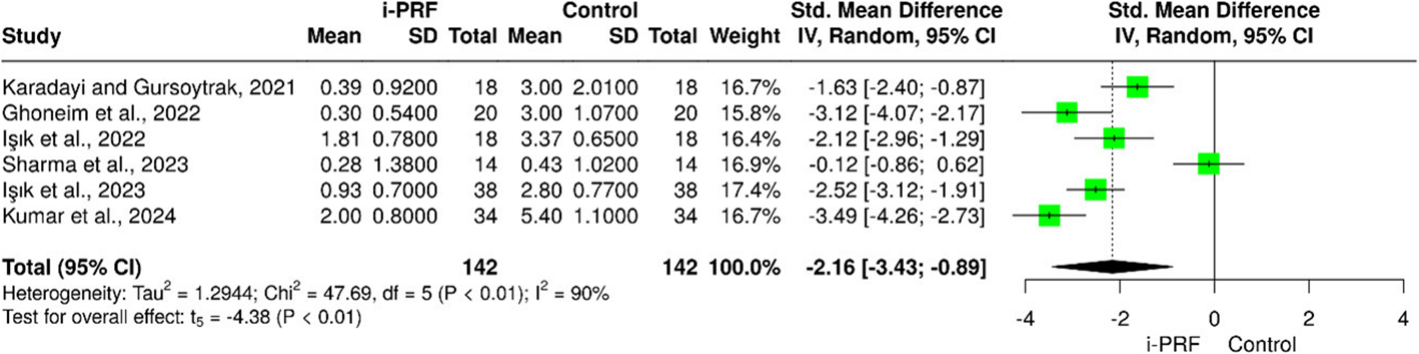
Forest plot for the i-PRF effect on TMJ pain

Six studies [25–30] evaluated changes in pain using the Visual Analogue Scale (VAS). Meta-analysis demonstrated that i-PRF resulted in a significantly greater reduction in pain compared with control interventions (pooled SMD = − 2.16; 95% CI = − 3.73 to − 0.59; *P* = 0.007). Notably, Işik et al. [27, 29] reported that, only in the control group, pain levels increased significantly from the 6th month to the 12th month. Considerable heterogeneity was observed among studies (I² = 86%), reflecting differences in patient selection, injection frequency, and follow-up duration. (Fig. 2).

### i-PRF effect on MMO

**Fig. 3:**
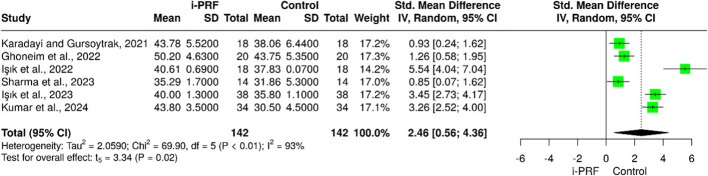
Forest plot for the i-PRF effect on MMO

Six studies [25–30] assessed improvements in MMO following treatment. Ghoneim et al. [26] reported a positive effect on MMO in both the i-PRF group and the control group. However, the i-PRF group showed higher values of MMO than that in the control group. Similar effect on MMO was observed by Karadayi et al. [25], and Işik et al. [27, 29] where i-PRF outperformed the other interventions used. Nevertheless, Karadayi et al. [25] found that in 3 months, the difference between the i-PRF group and the control group was negligible. In Kumar et al. [30] study, the authors documented a significant improvement in the i-PRF group compared to the control group who received no active treatment. Pooled analysis showed a significant mean increase of 2.46 mm in the i-PRF group compared with controls (95% CI = 0.56 to 4.36; *P* = 0.02). Considerable heterogeneity was also noted among studies (I² = 93%) (Fig. 3).

### i-PRF effect on protrusive & lateral mandibular movements

Four studies [25–29] noted the effect of i-PRF on lateral excursions while 3 studies [27–29] evaluated its effect on protrusive movements as well. In 2 studies by Işik et al. [27, 29], there was an increase in the range of protrusive and lateral movements over the 12 months of follow-up with a statistically significant difference at the 1 st and the 2nd month postoperatively. However, no significant difference was noted from the 2nd till the 12th month postoperatively. Furthermore, in the control group, the range of excursive movements decreased significantly from the 6th to 12th month.

Ghoneim et al. [26] reported a statistically significant increase in the measurements of lateral movements within each group over time where the difference between both groups was statistically significant.

**Fig. 4:**
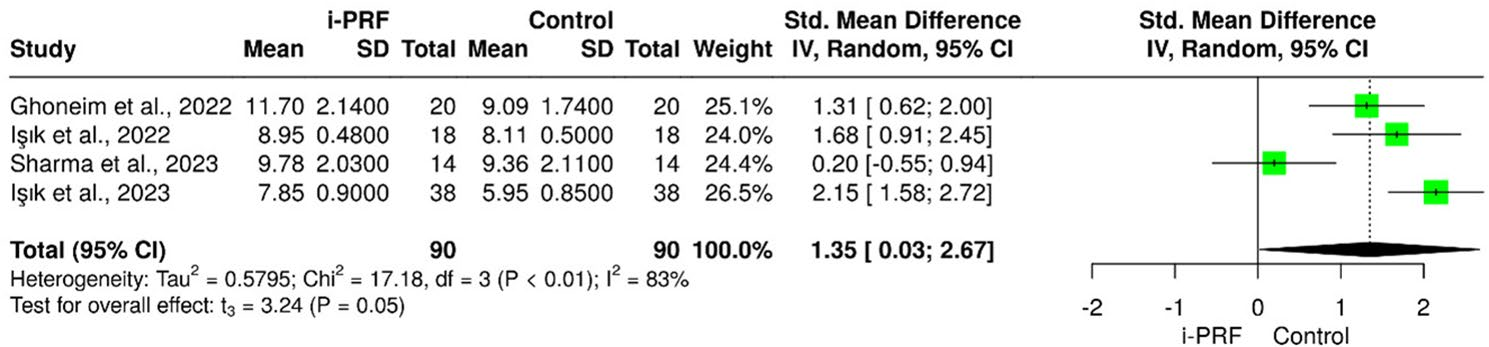
Forest plot for the i-PRF effect on lateral mandibular movements

**Fig. 5:**
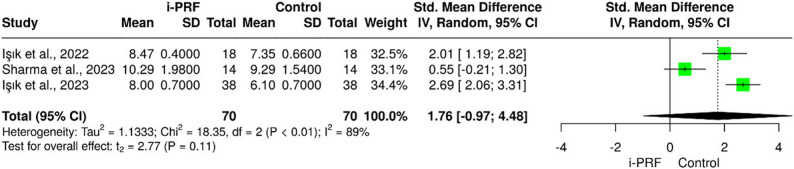
Forest plot for the i-PRF effect on protrusive mandibular movements

The pooled meta-analysis demonstrated a moderate overall effect favoring i-PRF for lateral movements (SMD = 1.35; 95% CI = 0.03 to 2.67; *P* = 0.048; I² = 83%), indicating substantial heterogeneity among studies (Fig. 4). For protrusive movements, the pooled effect was not statistically significant (SMD = 1.76; 95% CI = − 0.97 to 4.48; *P* = 0.11; I² = 89%), suggesting that improvements were inconsistent across trials. These findings indicate that i-PRF may contribute to short-term improvement in lateral mandibular movement, while its effect on protrusive movement remains uncertain due to inter-study variability and small sample sizes (Fig. 5).

### i-PRF effect on TMJ sounds

Three studies reported [25, 26, 30] on joint clicking or crepitation. Owing to variation in outcome reporting scales, quantitative synthesis was not feasible; however, the qualitative trend suggested symptomatic improvement. Ghoneim et al. [26] found that TMJ clicking ceased in 75% of the patients after 1-week post-injection in the i-PRF group. Moreover, there was a statistically significant reduction in TMJ clicking up to 6 months. In Sharma et al. study [28], TMJ clicking was present in 71.4% of the joints, in the i-PRF group, pre-injection. In the same study, a significant disappearance in TMJ clicking was noted in 2 weeks posttreatment and after 1 month from the first injection, clicking was found only in 35.7% and by the 5th month, no clicking was noted in all patients.

In U. Karadayi and B. Gursoytrak study [25], the Helkimo clinical dysfunction scoring was used where it includes joint sounds as a clinical criterion. However, no specific results on joint sounds were mentioned.

### i-PRF effect on disc position

Only one study [28] assessed the disc position on magnetic resonance imaging (MRI) where the authors found that disc position had improved toward normal with significant changes in both groups at the 9-month follow up; however, the i-PRF group showed better results.

### i-PRF effect on joint effusion

One study [28] addressed joint effusion in which it was found that the joint effusion was significantly less at the 9-month follow-up.

### Follow-up intervals

Five studies [26–30] out of the 6 included studies were long-term ones; in which Işik et al. studies [27, 29] had a 12-month follow-up period, Sharma et al. study [28] had a 9-month follow up, while Ghoneim et al. [26], and Kumar et al. [30] had a 6-month follow-up.

### Reported side effects

Five studies [25, 27–30] reported no adverse effects after i-PRF injections. However, in one study by Ghoneim et al. [26], two patients experienced transient inability to occlude in maximum intercuspation in the first 2 days after i-PRF injection.

#### Risk of bias within studies

**Fig. 6:**
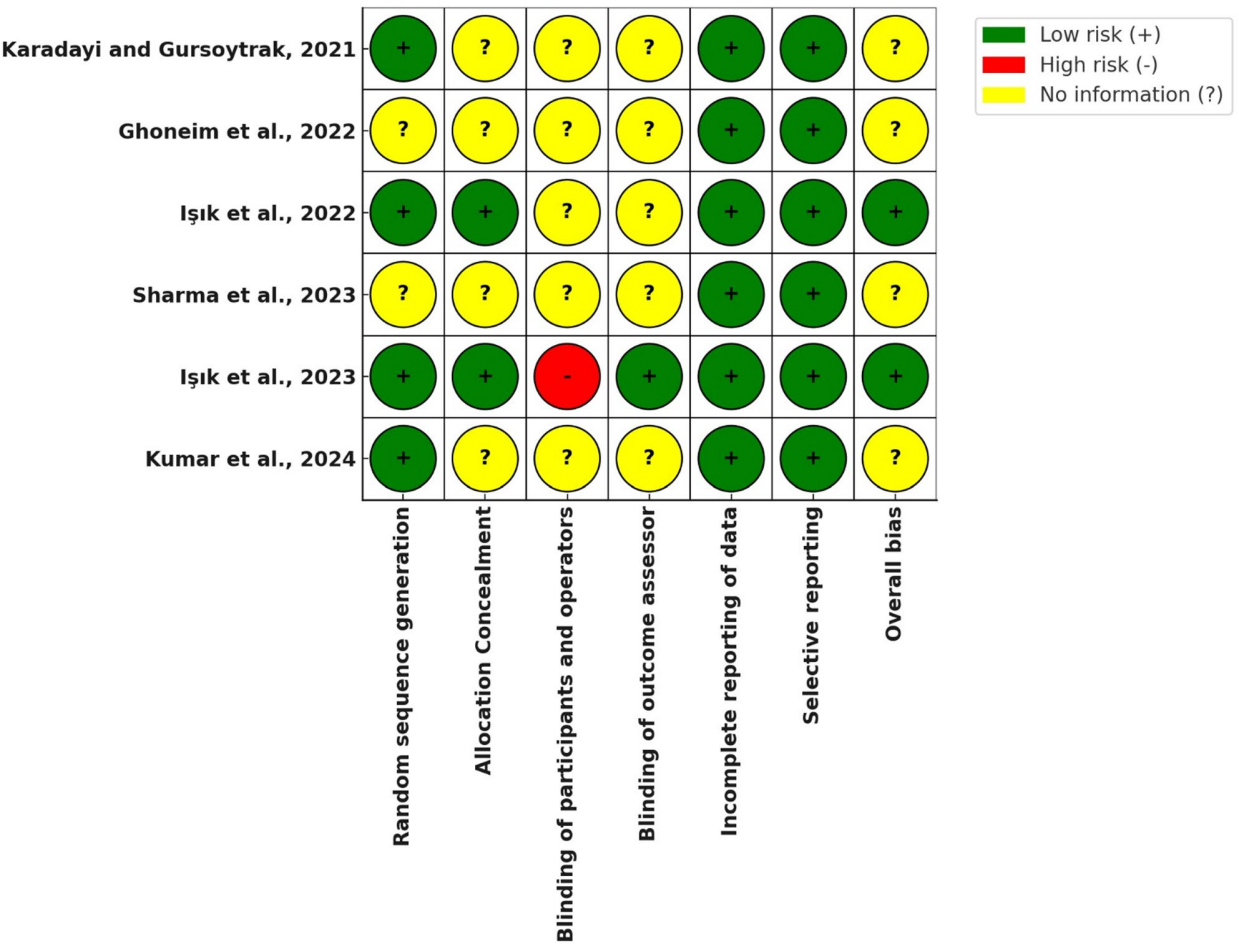
The risk of bias within studies

The risk of bias within studies, in compliance with the Cochrane Collaboration’s tool for assessing risk of bias, is shown in Fig. 6. The bias due to blinding was the most common throughout the studies.

## Discussion

Since it was introduced a decade ago, i-PRF has proved to have several advantages over PRP in the field of regenerative medicine [32–38]. Based on the beneficial effects of the i-PRF, it has gained attention as an intra-articular agent to treat intracapsular TMD. The current systematic review and meta-analysis aims to focus on the clinical evidence on the effects of the intra-articular injection of i-PRF for the management of patients suffering from either TMJ-ID or TMJ-OA.

This systematic review revealed promising results on the effectiveness of i-PRF in improving MMO, relief of pain as well as reducing joint clicking. Furthermore, in their retrospective short-term study, Torul et al. [22] stated that i-PRF after arthrocentesis led to reduction in pain scores and improvements in MMO and turned out to be of more effectiveness than arthrocentesis alone or with hyaluronic acid (HA). Similar findings were documented by Yuce E and Komerik N [23] in their cohort study. Moreover, Vingender et al. [31] stated that PC as i-PRF and RRP should be preferred as an intra-articular agent for the management of TMD.

In all the included RCTs [25–30], the injection of i-PRF in the superior joint compartment showed better results than the comparative treatment regarding pain levels, and MMO. These results go in accordance with Al-Delayme et al. [14], Torul et al. [22], Yuce et al. [23], Manafikhi et al. [24], and Vingender et al. [31] who reported similar effects of PC derivatives on TMD symptoms and MMO. Furthermore, Sharma et al. [28] reported noticeable changes toward the normal articular disc position with reduction in the joint effusion where the authors mentioned an improvement in the disc position in both study groups where PRP and i-PRF were injected in joints with disc displacement with reduction and without reduction as well. Similar effect was noted by Khallaf et al. [18] after the injection of PRP, another form of PC, where they mentioned a significant change towards the normal disc position. These promising findings may be due to the intra-articular regenerative injections into the joint spaces that enhance the micro-environment within the joint leading to the relief of TMD symptoms in a short period as well as prevention of further progression of the ID condition. Moreover, regenerative injections inhibit inflammatory biomarkers, produce more collagen fibrils, widen the joint space, and shift the disc mobility range owing to the in-situ growth factors [13, 32–38]. 

Sharma et al. [28] reported that clicking was found in five cases out of 7 patients after the 1 st injection of i-PRF while it was reduced to four after the 2nd injection then one (7.1%) at 3rd and 4th injection. By the 5th injection of i-PRF, no joint clicking was noted. This goes in agreement with Manafikhi et al. [24] prospective single-arm clinical study where they found that the clicking ceased in 70% of the patients after 1 week from receiving the 1 st injection of i-PRF, and in all the patients after 1 week from the 2nd injection. However, they reported that clicking returned to two of the patients after 6-month from the 1 st injection. On the other hand, Sharma et al. [28] found that clicking did not return up to the 9-month follow up. Moreover, this might be attributed to the improvement in the disc position reported by the same authors.

Ultrasound-guided intra-articular injections have been used in the past years, making the procedure safe and anticipated with minimum side effects [14, 17, 40–42]. Although all the intra-articular injections in the RCTs included in the present review were not guided by ultrasound, the treatment was effective with no major side effects. Only one study [26] reported a side effect where two patients experienced transient inability to occlude in maximum intercuspation in the first 48 h after the i-PRF injection. This might be contributed to the clotting of the liquid PRF.

The strengths of our systematic review include addressing a topic that has gained attention in regenerative medicine and the research field of TMDs with promising results. In addition to carrying out a meticulous methodology that integrates scientifically based research. However, there are some limitations such as the lack of blinding in most studies which introduces a risk of bias, and that a small number of RCTs were included owing to the lack of RCTs in literature addressing i-PRF effect on TMJ pain and joint dysfunction related to TMJ-ID and TMJ-OA. Moreover, the heterogeneity in the patient baseline characteristics, injection protocols, control groups, follow-up duration, assessment methods, and the fact that only one study [28] assessed disc position and joint effusion on MRI, limiting mechanistic insights pose as further shortcomings of the current review. Nevertheless, these limitations highlight the need for further long-term RCTs with standardized i-PRF preparation and injection protocols that may employ the use of ultrasound-guided intra-articular injections.

##  Conclusion

Promising results were found on the effect of i-PRF in the management of TMJ pain and joint dysfunction related to TMJ-ID and TMJ-OA. However, more RCTs are needed to further investigate its beneficial effects.

## Data Availability

All the dataset interpreted during the present review and meta-analysis are obtainable from the corresponding author upon rational request.

## Abbreviations

i-PRF: injectable Platelet Rich Fibrin
TMJ-ID: Temporomandibular Joint Internal Derangement
TMJ-OA: Temporomandibular Joint Osteoarthritis
PICO: Population, Intervention, Comparison, Outcome
RCTs: Randomized Clinical Trials
MMO: Maximum Mouth Opening
TMDs: Temporomandibular Disorders
TMJ: Temporomandibular Joint
SMD: Standardized Mean Difference (SMD)
PC: Platelet Concentrates
PRP: Platelet-Rich Plasma
MeSH: Medical Subject Headings
VAS: Visual Analogue Scale
HCDS: Helkimo Clinical Dysfunction Score
